# High-quality phased genome assemblies of line-bred Korean Hanwoo cattle

**DOI:** 10.1038/s41597-025-06069-3

**Published:** 2025-11-18

**Authors:** Jeong Woen Shin, Hyoun Ju Kim, Shil Jin, Yoonji Chung, Seung Hwan Lee, Jun Kim

**Affiliations:** 1 Department of Convergent Bioscience and Informatics, College of Bioscience and Biotechnology, Chungnam National University, Daejeon, Republic of Korea; 2 Planning&Coordination Division, National Institute of Animal Science, Rural Development Administration, Wanju-gun, Republic of Korea; 3 Hanwoo Research Center, National Institute of Animal Science, Rural Development Administration, Pyeongchang, Republic of Korea; 4 Institute of Agricultural Science, Chungnam National University, Daejeon, 34134 Republic of Korea; 5 Division of Animal & Dairy Science, Chungnam National University, Daejeon, 34134 Republic of Korea; 6 Graduate School of Life Sciences, College of Bioscience and Biotechnology, Chungnam National University, Daejeon, Republic of Korea

**Keywords:** Comparative genomics, Animal breeding

## Abstract

The Korean cattle breed, Hanwoo, has been selected as a meat cattle breed since the late 1980s in Korea, but has suffered from reduced genetic diversity due to repeated selection within a single population. In this study, we generated high-fidelity long-read sequencing data (~Q30, 98–146 Gb) for three Hanwoo Research Center (HRC) Hanwoo individuals from early, intermediate, and current breeding generations of the genetically distinct HRC population. These datasets yielded four partially phased genome assemblies of early and intermediate generations and two fully phased genome assemblies of the current generation. Furthermore, we construct a graphical pangenome reference by combining 19 publicly available cattle assemblies with our six new assemblies, identifying 39.3 M single-nucleotide variants (SNVs) and 60.7 K structural variants (SVs). Among these, 27.8 K SNVs and 26 SVs were uniquely found in the HRC Hanwoo population. These high-quality genomic resources provide valuable insights into the genetic characteristics of HRC Hanwoo and will facilitate future breeding strategies and genetic improvement efforts.

## Background & Summary

Cattle have long been domesticated, for more than 10,000 years, not only to provide labour for humans but also to supply nutrient-rich foods such as milk and meat^1^. Domestication began in Southwest Asia and spread to Europe along the migratory routes of pastoralists^2^. As a result, diverse breeds of cattle were established in different regions, influenced by their unique environmental and geographical characteristics^3^. Breeding has focused on reinforcing specific phenotypes suited to particular purposes, and through repeated breeding, specific traits in each population have been enhanced, leading to the accumulation of population-specific genetic variations^4^. However, research on various types of genetic variants in cattle breeds, including Hanwoo, remains limited.

Population-specific traits have been thoroughly investigated in cattle breeds based on accumulated data on linkage maps, genomes, transcriptomes, and QTLs^5–9^. However, prior to the availability of long-read sequencing technologies, most genetic resources were established by aligning short-read sequencing data to the ARS-UCD reference genome, which limited the discovery of population-specific structural variants (SVs; ≥50 bp) in addition to single-nucleotide variants (SNVs)^7,10–13^. Recent advances in long-read sequencing have now enabled not only high-quality *de novo* genome assemblies but also population-scale analyses of SVs, allowing the identification of SVs associated with quantitative trait loci (SV QTLs). Indeed, several studies have applied long-read sequencing to cattle cohorts and demonstrated the contribution of SVs to complex traits through SV QTL mapping^14–17^. In parallel, bovine pangenome projects have been established, which not only capture breed-specific genomic diversity but also successfully reveal trait-associated SVs with potential functional consequences^18–20^. These findings demonstrate that long-read–based analyses in cattle have already yielded population-scale SV QTL results and trait-associated SV discoveries, directly addressing gaps that could not be resolved by short-read sequencing approaches.

Hanwoo is one of the most representative cattle breeds in the Republic of Korea. Intensive breeding for meat-type traits began in the 1960s, and in 1987, the selection of Korean Proven Bulls (KPN) through progeny testing led to a more systematic breeding program^21^. Currently, semen from the KPN population is used to fertilise most of the Hanwoo population, which has accelerated the enhancement of economically important traits such as yearling weight and carcass weight^22,23^. However, the repeated use of the same semen has led to increased inbreeding and decreased genetic diversity within Hanwoo populations^22,23^. In the long term, this could undermine the breed’s industrial viability by exacerbating issues like disease outbreaks and reduced fertility, making it urgent to develop appropriate countermeasures.

Since 2009, the Hanwoo Research Center (HRC) at the National Institute of Animal Science (NIAS) has preserved the original genetic diversity of Hanwoo by establishing and maintaining a line-breeding Hanwoo population (HRC population)^24^. In a previous study, current HRC Hanwoo and KPN Hanwoo populations were compared based on microsatellites, revealing that the two populations are genetically distinct^24^. However, there remains a lack of comprehensive research on whether such differences can help restore genetic diversity or resolve productivity-related issues by providing beneficial genetic variants, specifically SVs. These SVs can be detected using diverse approaches. Short-read mapping-based approaches include Delly, Manta, GridSS, Wham, Canvas, whereas long-read mapping-based tools such as Sniffles, SVIM, pbsv, and cuteSV have been widely applied^25–33^. These mapping-based computational pipelines remain effective even under low sequencing coverage. Nevertheless, when high-coverage long-read sequencing data are generated, it is generally more appropriate to perform SV calling on the assembled genome rather than directly on the reads^34^. Several established tools, including dipcall, SVIM-asm, PanGenome Graph Builder, and minigraph-cactus, have been widely used to enable such assembly-based SV calling, with minigraph-cactus emerging as one of the most widely adapted methods in recent studies^32,35–39^.

The recently released long-read sequencing-based Hanwoo *de novo* genome assembly (SNU_Hanwoo_2.0) offers valuable insights into Hanwoo-specific SVs, yet it still has several limitations^40^. First, SNU_Hanwoo_2.0 was partially phased using 24.8 × long-read sequencing data from a single individual without any parental genomic information, and only one of its haplotypes is publicly available. This makes it difficult to determine which haplotype detected variants belong to and how they are linked. Second, the sample used for SNU_Hanwoo_2.0 is a Hanwoo individual whose sire was from the KPN population, suggesting that it may carry only a portion of the genetic variations present in the original Hanwoo population and may have suffered from inbreeding depression. As a result, it is challenging to discern the zygosity of the detected variants and whether they became homozygous due to phenotype-based selection or if they arose from increased inbreeding. Third, the HiFi data used for the genome assembly was processed with PacBio CCS version 6.3.0, which was before the integration of the deep learning-based DeepConsensus model, later introduced in version 7.0 on the Revio platform^41^. Finally, no short-read sequencing data for the same individual is available, which is important for measuring base-level quality metrics.

**Fig. 1:**
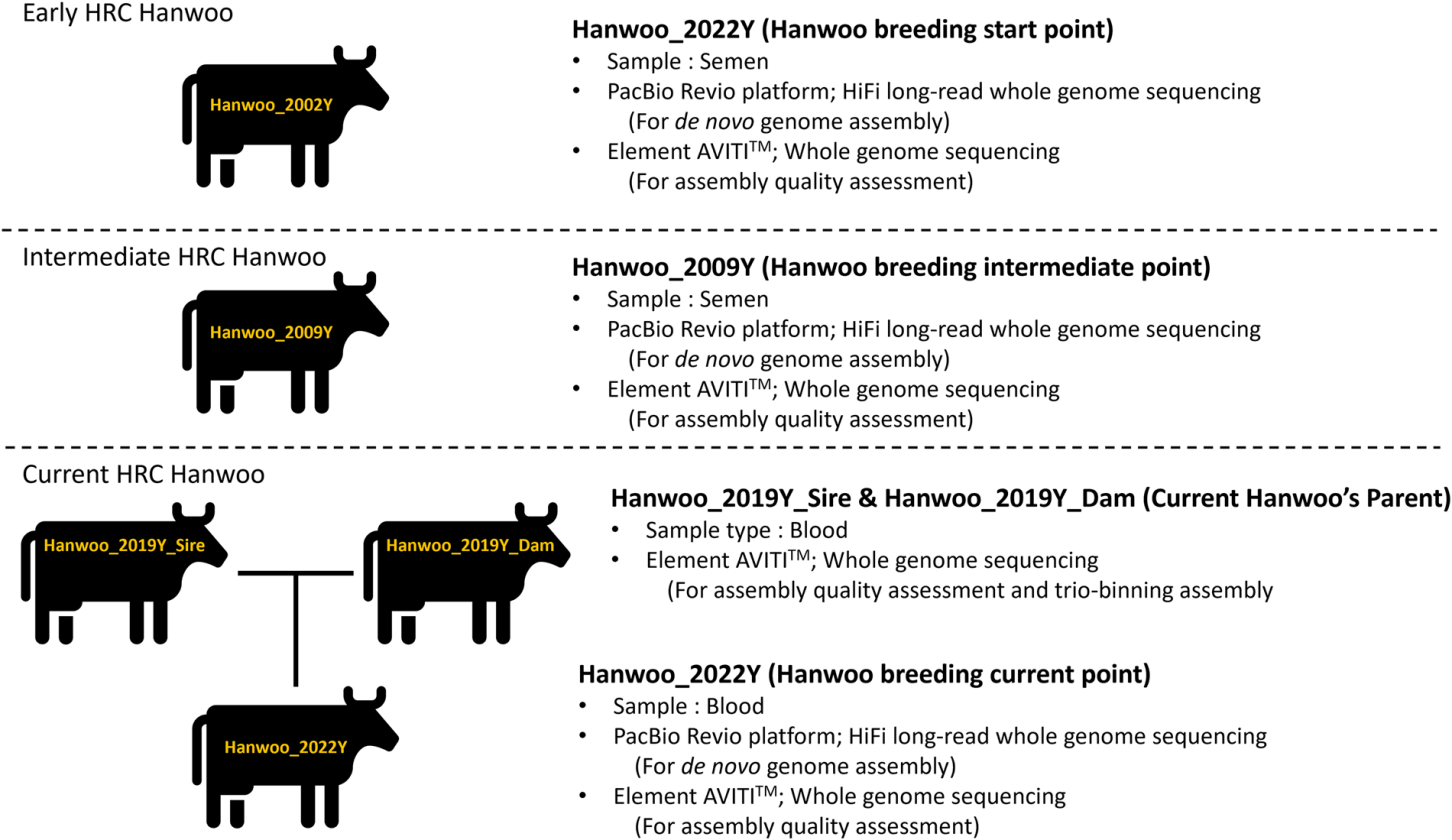
Schematic representation of this study. In this study, we generated both long-read and short-read sequencing data from three HRC Hanwoo individuals representing different time periods to construct a graphical genome assembly of Hanwoo. Additionally, we produced short-read sequencing data from both parents of a current Hanwoo individual to enable the construction of two fully phased genome assemblies. The quality of each assembly was then evaluated using the corresponding short-read sequencing data.

To overcome these limitations, this study aimed to obtain high-quality genome assemblies from three HRC Hanwoo individuals that preserve the genetic diversity of the early Hanwoo population. Specifically, we sought to obtain approximately 60× coverage of PacBio HiFi sequencing data with the DeepConsensus base-calling model for three representative individuals from early (2002), intermediate (2009), and current (2019) breeding generations, with the objective of enhancing assembly contiguity. In addition, we further aimed to generate two fully phased genome assemblies by sequencing both parents of the current individual. Also, we produced matched short-read sequencing data for each individual to validate assembly quality. By integrating the newly constructed Hanwoo genome assemblies with previously published genome assemblies from various cattle species, we aimed to construct a graph-based pangenome. Through this approach, our objective was to identify total Hanwoo-specific and HRC-specific genetic variants (Fig. 1). We expect that these high-quality Hanwoo genome assemblies will serve as critical references for maintaining population quality and improving traits.

## Methods

### Hanwoo sampling

All Hanwoo samples used in the study were bred and collected by HRC. Our three line-breeding male Hanwoo individuals born in 2002 (Hanwoo_2002Y), 2009 (Hanwoo_2009Y) and 2022 (Hanwoo_2022Y), were randomly selected. Additionally, the parents of Hanwoo_2022Y (Hanwoo_2019Y_Sire and Hanwoo_2019Y_Dam) were also utilized in this study. The semen extracted from Hanwoo_2002Y and Hanwoo_2009Y was used for sequencing, which had been aliquoted into straws, pre-frozen, and stored in liquid nitrogen at −196 °C. The blood samples from Hanwoo_2019Y_Sire, Hanwoo_2019Y_Dam, and Hanwoo_2022Y were used for sequencing, which were collected and stored at −80 °C after freezing.

### DNA extraction and whole-genome sequencing

DNA extraction and whole-genome sequencing were performed by Eyeoncell Co., Ltd. Specifically, genomic DNA was extracted from semen samples following the PLOS ONE Lab Protocols (https://www.protocols.io/view/dna-isolation-from-cattle-semen-for-long-read-sequ-j8nlkw1qwl5r/v1), using all specified reagents except for Proteinase K (NEB, P8107S). For blood samples, genomic DNA was isolated using the Quick-DNA HMW MagBead Kit (Zymo Research, D6060).

For short-read sequencing data of five Hanwoo individuals, genomic DNA was processed to obtain sequencing libraries using the Elevate Enzymatic Library Prep Kit (#830-00009) and the Elevate Long UDI Adapter Kit Set A (#830-00010). The prepared DNA libraries were sequenced using the AVITI sequencer, producing 151-bp paired-end reads.

Additionally, PacBio HiFi data were produced using genomic DNA from three Hanwoo individuals, Hanwoo_2002Y, Hanwoo_2009Y, and Hanwoo_2022Y. DNA fragments smaller than 10 kb were removed using the Short Read Eliminator (SRE) Kit (102-208-300), and the resulting genomic DNA was sheared with a Covaris g-TUBE and cleaned. The sheared DNA was then processed using the SMRTbell Prep Kit 3.0 (102-166-600) to construct PacBio sequencing libraries, and the final libraries were sequenced on the PacBio Revio platform.

The short-read sequencing data yielded 76.75 Gb to 140.47 Gb, while the PacBio HiFi data produced 176.51 Gb to 196.01 Gb. Assuming a Hanwoo genome size to be 3.1 Gb, the short-read sequencing data achieved a high coverage of 31.68× to 47.26×, and the PacBio HiFi data showed 56.94× to 63.23× coverage, sufficient for comprehensive genome analysis (Table 1). All sequencing data have been uploaded to the NCBI Sequence Read Archive under accession SRP547596^42^.

**Table 1:** Statistics of raw genomic sequencing data.

platform	sample	birth year	sex	num_seqs	sum_len	coverage(x)	minimum length	average length	maximum length	Q1	Q2	Q3	N50	N50_num	Q20(%)	Q30(%)	GC(%)	AvgQual	GC(%)
AVITI	Hanwoo_2002Y	2002	M	846,780,990	127,017,148,500	40.97	150	150	150	150	150	150	150	1	95.41	89.04	43.7	23.03	43.7
AVITI	Hanwoo_2009Y	2009	M	936,467,252	140,470,087,800	45.31	150	150	150	150	150	150	150	1	95.44	89.3	44.14	22.52	44.07
AVITI	Hanwoo_2019Y_Sire	2019	M	976,644,098	146,496,614,700	47.26	150	150	150	150	150	150	150	1	96.81	92.32	43.98	22.36	43.43
AVITI	Hanwoo_2019Y_Dam	2019	F	654,632,760	98,194,914,000	31.68	150	150	150	150	150	150	150	1	96.18	90.92	43.7	22.25	44.14
AVITI	Hanwoo_2022Y	2022	M	786,968,246	118,045,236,900	38.08	150	150	150	150	150	150	150	1	95.48	89.16	43.43	22.29	43.7
PacBio	Hanwoo_2002Y	2002	M	11,774,158	183,093,270,413	59.06	143	15,550.40	65,822.00	12,073.00	15,244.00	18,894.00	16,982.00	22,500.00	97.49	93.97	42.59	24.38	42.81
PacBio	Hanwoo_2009Y	2009	M	10,906,265	176,512,698,428	56.94	119	16,184.50	74,182.00	12,619.00	15,712.00	19,508.00	17,462.00	25,568.00	97.45	93.87	42.51	25.1	42.51
PacBio	Hanwoo_2022Y	2022	M	12,097,908	196,011,892,998	63.23	70	16,202.10	64,053.00	11,928.00	15,821.00	20,213.00	18,220.00	27,065.00	96.97	92.77	42.81	25.17	42.59

**Fig. 2:**
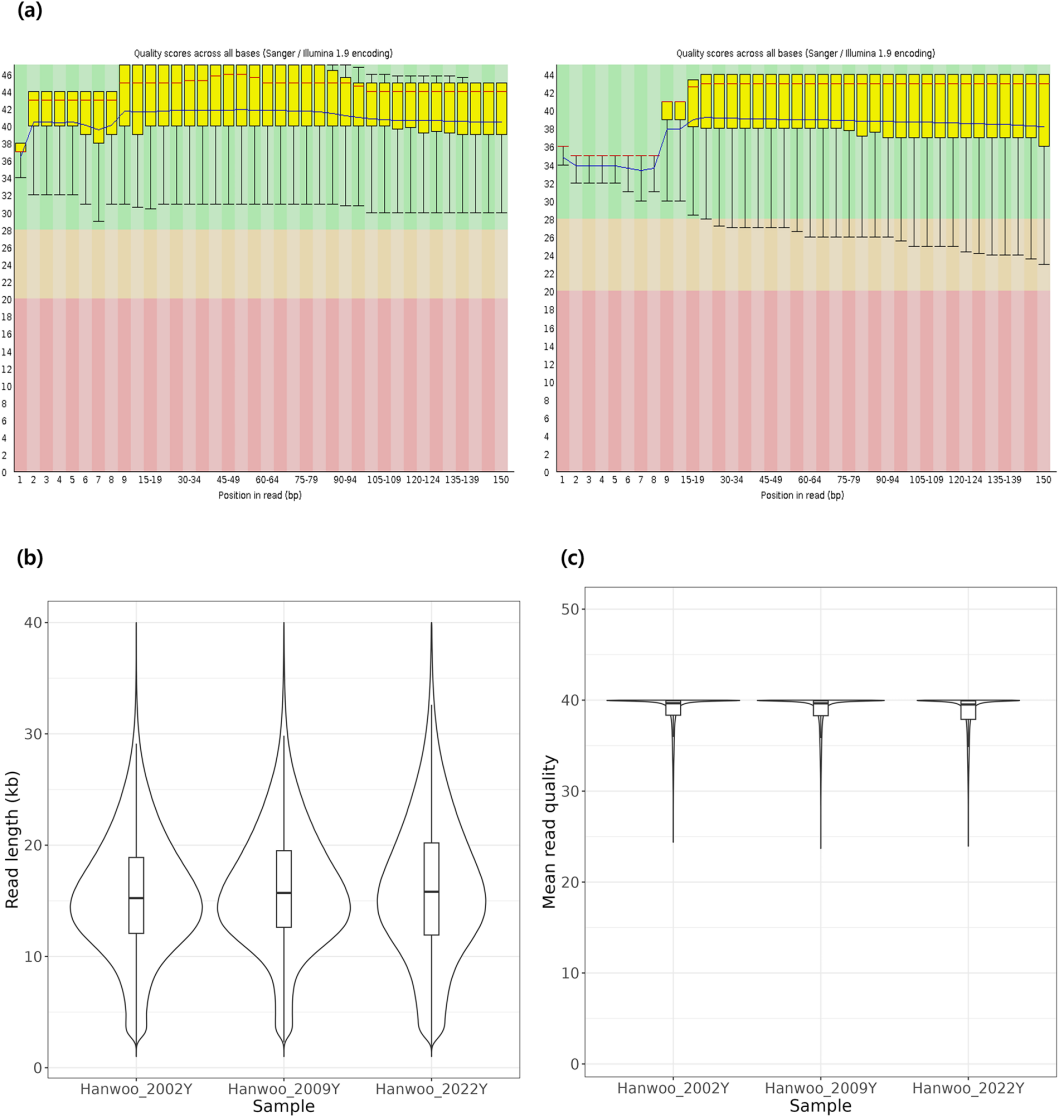
Assessing quality of short- and long-read sequencing data. *a*, Base quality scores across all positions in the short-read sequencing data, assessed using FastQC. The x-axis represents the position in the read, and the y-axis shows the base quality score. Yellow boxplots indicate the quality distribution at each position, with the red line representing the mean. Green shaded areas denote high-quality scores. As the data are paired-end, two plots are shown per sample. *b*, Distribution of PacBio HiFi read lengths per individual. The x-axis indicates each individual, and the y-axis shows the length of the reads. *c*, Quality score distribution of PacBio HiFi reads. Read quality scores are plotted on the y-axis, grouped by individual on the x-axis.

To assess the quality of the raw data, FastQC (version 0.12.1; *fastqc -o ${PREFIX} -f fastq ${FASTQ_READ1} ${FASTQ_READ2}*) and bioawk (version 20110810; *bioawk -c fastx -v PREFIX = “${PREFIX}” ‘OFS = “,”{print PREFIX, length($seq), meanqual($qual)}’*) were used for the short-read and long-read sequencing data, respectively^43,44^. The short-read sequencing data exhibited high per-base quality across all reads (Fig. 2a). Also, the PacBio HiFi data showed an average read length of 15.55 Kb to 16.20 Kb, with more than 74% of reads per sample exhibiting a quality score of Q38 or higher, underscoring the high fidelity of the data in terms of both read length and base-calling accuracy (Fig. 2b–c).

### Genome assembly and quality assessment

For the Hanwoo_2002Y and Hanwoo_2009Y samples, PacBio HiFi reads were assembled to the contig level using hifiasm (version 0.19.8-r603; *hifiasm -o ${PREFIX} ${HiFi}--hg-size 3.1 g*)^45^. For the Hanwoo_2022Y sample, a parental *k*-mer database was generated using yak (version 1.0; yak count -b 37 -o ${PREFIX}.yak < (zcat ${MATERNAL or PATERNAL}*fastq.gz) < (zcat ${MATERNAL or PATERNAL}*fastq.gz)), and the contig-level trio-binning assembly was performed using hifiasm (version 0.19.8-r603; *hifiasm -o ${prefix}.trio -1./yak/${Paternal}.yak -2./yak/${Maternal}.yak ${HiFi}*)^45^. These genome assemblies have been deposited in NCBI GenBank and are publicly accessible^46–51^.

To assess the quality of the contig-level assembly, a *k*-mer database for the matched short-read data was created using meryl (version 1.3; *meryl k* = *${K} memory* = *${MEMORY} threads* = ${THREADS} count ${SAMPLE}.*.fq.gz output ${SAMPLE}.db.meryl)^52^. In the case of the Hanwoo_2022Y assembly, additional parental short-read data were processed to generate their respective *k*-mer databases, and a hapmer database for each parent were also constructed using the hapmers.sh script from merqury (version 1.3; *hapmers.sh ${MATERNAL}.db.meryl ${PATERNAL}.db.meryl ${CHILD}.db.meryl*)^53^. Subsequently, phasing completeness and quality values (QVs) for the six genome assemblies were calculated with the meryl and hapmer databases using merqury (version 1.3; *merqury.sh ${CHILD}.db.meryl ${MATERNAL}.db.hapmer.meryl ${PATERNAL}.db.hapmer.meryl ${MATERNAL_ASSEMBLY} ${PATERNAL_ASSEMBLY} ${PREFIX}*)^53^.

As a result, the QVs for the six assemblies ranged from QV51.54 to QV53.08, emphasizing their high base-level accuracy. For Hanwoo_2022Y, the phasing completeness of the maternal and paternal haplotypes were measured as 99.07% and 96.49%, respectively, suggesting accurate phasing of diploid genome assemblies. The slightly lower *k-mer* completeness in the paternal haplotype is likely attributable to the absence of one X chromosome, which can reduce k-mer based completeness estimates and does not reflect an actual reduction in assembly quality. It is noteworthy that for the Hanwoo_2002Y and Hanwoo_2009Y samples, phasing completeness could not be calculated due to the absence of parental short-read sequencing data. For the public Hanwoo genome assembly, SNU_Hanwoo_2.0, we were unable to obtain matched and its parental short-read sequencing data, which hinders to assess the genome quality in terms of QVs and phasing completeness.

**Fig. 3:**
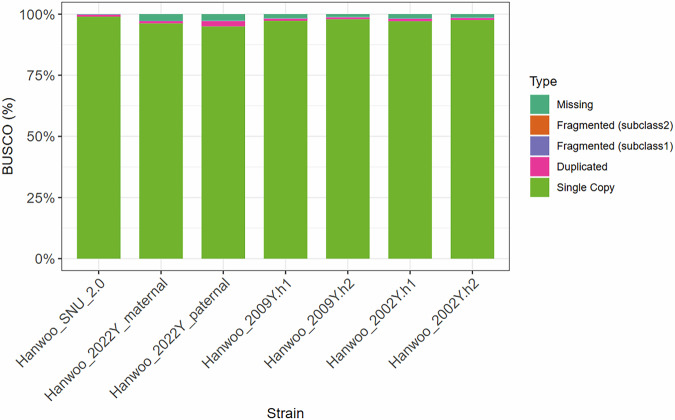
BUSCO completeness for Hanwoo assemblies. BUSCO completeness is classified into complete single-copy genes, duplicated complete genes, fragmented genes, and missing genes, with each category represented by a different color. Fragmented genes are further divided into two subclasses: subclass 1 refers to BUSCO genes of which only a portion is present in the assembly and the remainder cannot be aligned, while subclass 2 refers to BUSCO genes where different portions align to separate positions within the assembly. The x-axis denotes the assemblies, and the y-axis shows the cumulative proportion.

Additionally, we evaluated another completeness metric, single-copy orthologs completeness, using compleasm.py (version 0.2.7; *python compleasm.py run -a ${ASSEMBLY} --o ${PREFIX}–autolineage*)^54^. This Benchmarking Universal Single-Copy Orthologue (BUSCO) analysis with compleasm was performed based on the *artiodactyla_odb12* dataset^55,56^. The results showed that the proportion of complete single-copy genes in the six assemblies all exceeded 95%, indicating very high completeness (Fig. 3).

To construct a pangenome for cattle, we utilized a total of 19 chromosome-level genome assemblies available on the NCBI database. These assemblies represent 15 cattle breeds: Holstein-Friesian^57^, Jersey^58^, *Bos taurus* × *Bison bison*^59^, Mongolian^60^, *Bos grunniens* × *Bos taurus*^61,62^, Hanwoo^63^, Norwegian Red Cattle^64^, African Ankole cattle^65^, African N’Dama cattle^66^, Angus × Brahman F_1_ hybrid^67,68^, Tuli^69^, Wagyu^70^, Yunling cattle^71^, Hereford^72^ and Charolais^73^. These assemblies had been generated using various sequencing technologies such as PacBio, Oxford Nanopore Technologies, and Sanger platforms.

**Fig. 4:**
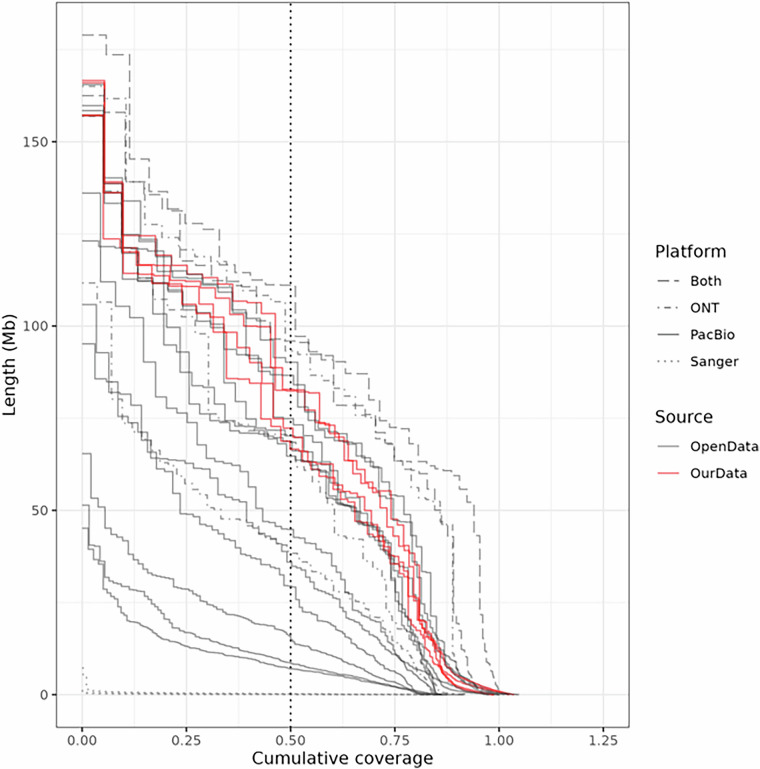
NGx plot for contig-level assemblies. Contigs are sorted in descending order by length; the y-axis represents the length of each contig, and the x-axis indicates cumulative coverage across contigs. Assemblies from our three HRC Hanwoo samples are shown in red, while assemblies from public datasets are shown in grey. Line types correspond to the long-read sequencing platforms used for each assembly. ONT: Oxford Nanopore Technologies. PacBio: Pacific Biosciences.

In order to avoid scaffolding errors, all assemblies except for ARS-UCD 2.0 were converted from chromosome-level to contig-level using bioawk (version 20110810; *bioawk -c fastx ‘{print $seq}’ | sed -r ‘s/n + /\n/gi’ | cat -n | awk ‘{print “ > ctg”$1; print $2}’*)^44^. Assembly statistics for a total 24 contig-level assemblies, including our 6 genome assemblies, were calculated using assembly-stats (version 1.01; *assembly-stats ${ASSEMBLY}*)^74^. The total length of each public genome assembly varied between 2.57 Gb and 3.24 Gb, with N50 values ranging from 0.96 Mb to 111.09 Mb (Table 2). Our genome assemblies exhibited a total length of 3.06 Gb to 3.21 Gb and N50 of 68.78 Mb to 90.19 Mb, suggesting higher contiguity. Cumulative coverage plots for the contig-level assemblies were visualized using the ggplot2 package in R program (Fig. 4)^75^.

**Table 2:** Assembly statistics of the 24 cattle genome assemblies.

Assembly name	GenBank ID	Submitter	Date	Breed	Tissue	Sequencing technology	sum (bp)	contig number	average (bp)	largest (bp)	N50
ARS-LIC_NZ_Holstein-Friesian_1	GCA_021347905.1	USDA ARS	1/11/2022	Holstein-Friesian	blood	PacBio RSII	2,665,138,195	3,129	851,754	45,157,408	8,737,306 (n = 90)
ARS-LIC_NZ_Jersey	GCA_021234555.1	USDA ARS	12/20/2021	Jersey	blood	PacBio Sequel	2,641,709,256	365	7,237,560	136,078,146	50,551,513 (n = 17)
ARS_Simm1.0	GCA_018282465.1	USDA ARS	5/6/2021	Bos taurus × Bison bison	lung	Oxford Nanopore	2,861,673,644	1,374	2,082,732	156,884,481	70,612,029 (n = 15)
ASM3988117v1	GCA_039881175.1	Northwest A&F University	5/29/2024	Mongolian	blood	Oxford Nanopore	2,637,224,782	697	3,783,680	111,682,347	43,517,586 (n = 22)
Btau_5.0.1	GCA_000003205.6	Cattle Genome Sequencing International Consortium	11/19/2015	Hereford	blood	Sanger; PacBio RS II	2,713,028,855	44,658	60,751	7,228,988	260,469 (n = 3,033)
ARS_UNL_Btau-highland_paternal_1.0_alt	GCA_009493655.1	University of Nebraska - Lincoln	11/4/2019	Bos grunniens × Bos taurus	lung	PacBio Sequel	2,668,830,262	473	5,642,347	157,044,929	71,681,919 (n = 14)
ARS_UNL_BGru_maternal_1.0_p	GCA_009493645.1	University of Nebraska - Lincoln	11/4/2019	Bos grunniens × Bos taurus	lung	PacBio Sequel	2,677,129,979	596	4,491,829	157,220,985	72,336,424 (n = 13)
SNU_Hanwoo_2.0	GCA_028973685.2	Seoul National University	9/22/2023	Hanwoo	blood	PacBio Sequel	3,108,480,684	1,720	1,807,256	123,092,485	64,712,170 (n = 18)
NRF	GCA_963921495.1	NORWEGIAN UNIVERSITY OF LIFE SCIENCES	6/26/2024	Norwegian Red Cattle	blood	Oxford Nanopore	3,190,064,063	716	4,455,397	165,017,368	93,255,103 (n = 13)
ROSLIN_BTI_ANK1	GCA_905123885.1	THE ROSLIN INSTITUTE	2/18/2022	African Ankole cattle	blood	PacBio Sequel	2,834,561,117	8,484	334,107	65,391,290	18,610,951 (n = 50)
ROSLIN_BTT_NDA1	GCA_905123515.1	THE ROSLIN INSTITUTE	2/18/2022	African N’Dama cattle	blood	PacBio Sequel	2,708,415,509	3,634	745,299	51,413,324	10,726,776 (n = 73)
UOA_Angus_1	GCA_003369685.2	University of Adelaide	11/30/2018	Angus × Brahman F1 hybrid	lung	PacBio RSII; PacBio Sequel	2,574,284,011	1,712	1,503,671	95,169,666	37,061,043 (n = 23)
UOA_Brahman_1	GCF_003369695.1	University of Adelaide	11/30/2018	Angus × Brahman F1 hybrid	lung	PacBio RSII; PacBio Sequel	2,679,316,559	1,552	1,726,364	79,234,837	26,764,281 (n = 32)
UOA_Tuli_1	GCA_040285425.1	University of Adelaide	6/25/2024	Tuli	blood	PacBio Sequel; Oxford Nanopore	3,033,659,093	909	3,337,359	162,485,535	95,916,531 (n = 13)
UOA_Wagyu_1	GCA_040286185.1	University of Adelaide	6/25/2024	Wagyu	blood	PacBio Sequel; Oxford Nanopore	3,141,302,879	419	7,497,143	178,935,652	111,091,738 (n = 12)
YAU_Btau_1.0	GCA_034097375.1	Yunnan Agricultural University	12/5/2023	Yunling cattle	Heart	PacBio Sequel	2,713,028,855	44,658	60,751	7,228,988	260,469 (n = 3,033)
Bos_taurus_UMD_3.1.1	GCA_000003055.5	Center for Bioinformatics and Computational Biology, University of Maryland	11/25/2014	Hereford	blood	Sanger	2,649,399,379	77,608	34,138	1,160,130	96,019 (n = 8,018)
seqoccin.Bt.char.v1.0	GCA_947034695.1	INRAE	10/29/2022	Charolais	blood	PacBio HiFi	3,244,632,666	1,444	2,246,976	158,432,915	84,059,894 (n = 15)
Hanwoo_2002Y.h1	GCA_049634445.1	Chungnam National University	4/15/2025	Hanwoo	semen	PacBio Revio	3,209,457,798	666	4,819,006	166,009,136	82,687,382 (n = 15)
Hanwoo_2002Y.h2	GCA_049634545.1	Chungnam National University	4/15/2025	Hanwoo	semen	PacBio Revio	3,059,190,886	756	4,046,549	157,106,588	82,383,052 (n = 14)
Hanwoo_2009Y.h1	GCA_049634505.1	Chungnam National University	4/15/2025	Hanwoo	semen	PacBio Revio	3,060,440,562	975	3,138,913	166,629,150	68,783,667 (n = 15)
Hanwoo_2009Y.h2	GCA_049634715.1	Chungnam National University	4/15/2025	Hanwoo	semen	PacBio Revio	3,174,661,532	641	4,952,670	157,175,098	69,826,876 (n = 16)
Hanwoo_2022Y_paternal	GCA_049634565.1	Chungnam National University	4/15/2025	Hanwoo	blood	PacBio Revio	3,176,365,834	611	5,198,635	165,496,400	73,191,946 (n = 16)
Hanwoo_2022Y_maternal	GCA_049634675.1	Chungnam National University	4/15/2025	Hanwoo	blood	PacBio Revio	3,099,434,866	457	6,782,133	159,797,483	90,185,745 (n = 14)

### Pangenome-level variant calling

By integrating 24 contig-level assemblies with the reference genome, ARS-UCD2.0, a graphical pangenome was constructed using minigraph-cactus (version 7.0.0 venv-cactus-v2.8.4; *cactus-pangenome ./jobstorepath ./sequenceFile.tsv --outDir ${PREFIX} –outName ${PREFIX} --reference ${REF_GENOME} --filter 2 --giraffe clip filter --vcf --viz --odgi --chrom --vg clip filter --chrom --og --gbz clip filter full --gfa clip full --vcf --giraffe --gfa --gbz --chrom-vg*)^76^. We only analysed common variants that were defined as those with a single alternative allele. As a result, total 47,303,284 common variants were identified, including 39,306,737 SNVs in addition to 8,686 deletion SVs and 52,034 insertion SVs.

We identified variants that were fixed across total four Hanwoo individuals, which were 1 KPN and 3 HRC individuals that have genome assemblies. A total of 10,036,782 non-missing common variants were genotyped, among which 732,314 SNVs, 124 deletion SVs, and 589 insertion SVs were found to be fixed in Hanwoo population. Of these variants, 530,798 variants were identified exclusively within three HRC population, comprising 530,208 SNVs, 75 deletion SVs, and 515 insertion SVs (Table 3).

**Table 3:** The number of Hanwoo- and HRC Hanwoo-specific variants.

Group	SNP	Deletion	Insertion
Hanwoo-specific variants	10,335	1	13
HRC-specific variants	27,858	5	21
Hanwoo-fixed variants	732,314	124	589
HRC-fixed variants	530,208	75	515

**Fig. 5:**
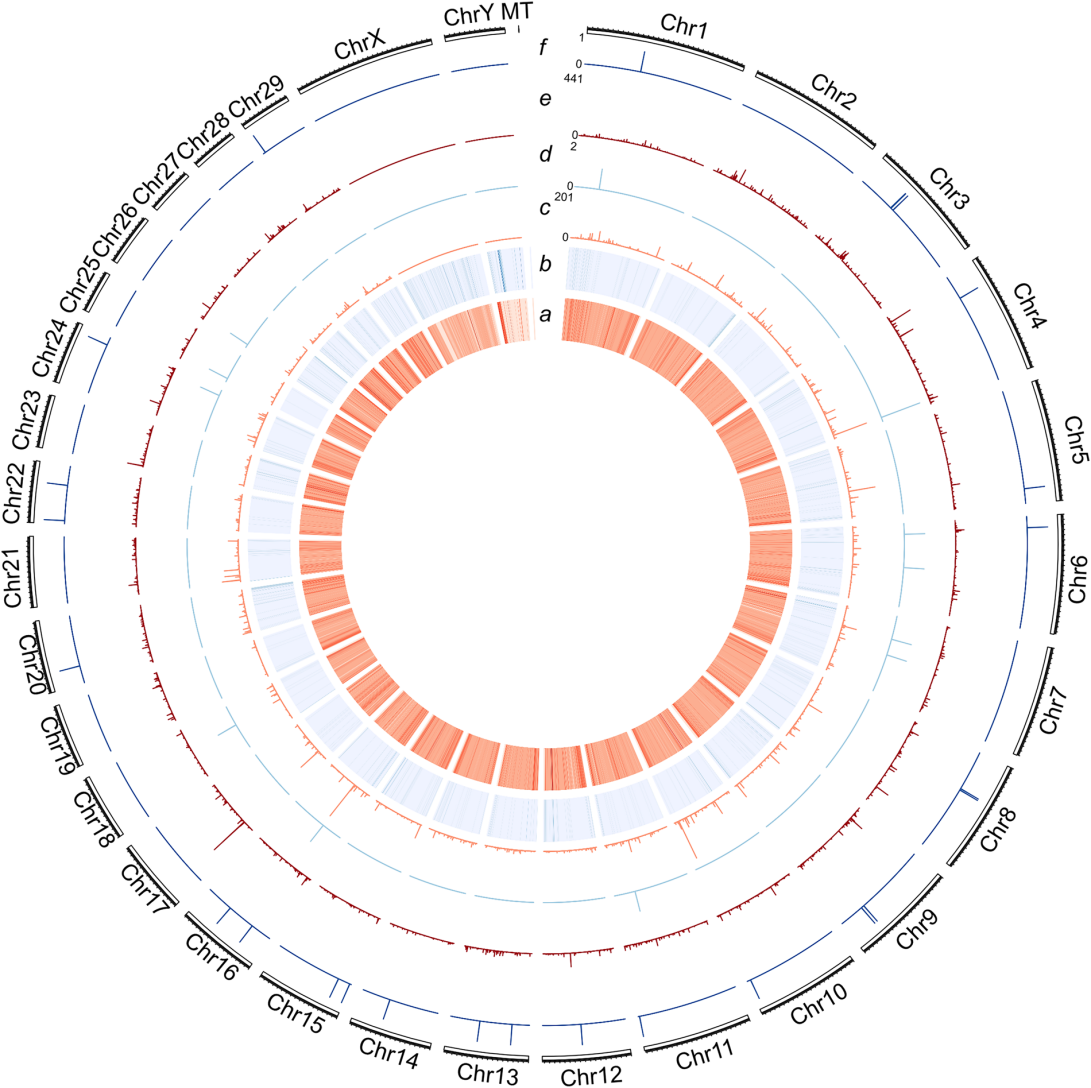
Circos plot of Hanwoo- and HRC Hanwoo-specific variants. SNV distributions are visualized as dot plots, and SVs longer than 50 bp are shown as bar plots along each chromosome. (**a**) Total SNVs identified from the pangenome (Maximum bin count: 8,908); (**b**) Total SVs identified from the pangenome (Maximum bin count: 108); (**c**) total Hanwoo-specific SNVs; (**d**), total Hanwoo-specific SVs; (**e**) HRC Hanwoo-specific SNVs; (**f**) HRC Hanwoo-specific SVs.

In addition, Hanwoo-specific variants were extracted by filtering out variants where all genotypes were the reference type in all non-Hanwoo samples, and HRC-specific variants were further filtered based on the previous KPN Hanwoo genome and our genome assemblies. It results in the identification of 10,335 SNVs, 1 deletion SV, and 13 insertion SVs as Hanwoo-specific variants, specifically 27,858 SNVs, 5 deletion SVs, and 21 insertion SVs as HRC Hanwoo-specific variants (Table 3). Chromosomal distributions of these Hanwoo-specific and HRC Hanwoo-specific variants were visualized using circos (version 0.69-8) (Fig. 5).

Although these results provide initial insights into Hanwoo- and HRC-specific variants, such findings should be interpreted with caution, as comprehensive breed- and line-specific comparisons require population-level analysis with sequencing data from a sufficiently large number of individuals. We anticipate that the accumulation of large-scale long-read sequencing datasets from many individuals will enable more refined and robust analysis in the future.

### Ethical statement

This study was approved by the Institutional Animal Care and Use Committee (IACUC) of the National Institute of Animal Science (NIAS; approval no. NIAS2019-0360 and NIAS 2025-0008). All procedures involving the handling and treatment of Hanwoo complied with the guidelines of the Hanwoo Research Center (NIAS). Experimental management was conducted in strict accordance with these regulations to ensure animal welfare and humane treatment, with all possible measures taken to minimize stress or discomfort. The study is reported in accordance with the ARRIVE guidelines to ensure transparent and comprehensive reporting.

## Data Records

Our genome assemblies and raw PacBio reads in FASTQ format were submitted to the NCBI BioProject database (https://www.ncbi.nlm.nih.gov/bioproject) under the accession number PRJNA1308631. All reads are available under SRP547596^42^, and genome assemblies are accessible as follows:

JBMUHS000000000 (GCA_049634445.1; haplotype1)^46^ and JBMUHR000000000 (GCA_049634545.1; haplotype2)^47^ for Hanwoo_2002Y, JBMUHU000000000 (GCA_049634505.1; haplotype1)^48^ and JBMUHT000000000 (GCA_049634715.1; haplotype2)^49^ for Hanwoo_2009Y; and JBMUHV000000000 (GCA_049634565.1; paternal)^50^ and JBMUHW000000000 (GCA_049634675.1; maternal)^51^ for Hanwoo_2022Y. Also, the variant data was submitted to the European Variant Archive (https://www.ebi.ac.uk/eva/) under the accession number ERP180032^77,78^.

## Technical Validation

Assembly quality was assessed using total size, contig length, and quality values. Total length of the 6 assemblies ranged 3.06–3.18 Gb and the N50 length were longer than 68.78 Mb. Quality values were about 53.

## Data Availability

All programs and pipelines were executed following their official manuals or help pages. Version and parameter information that we used for our analysis have been described in the Methods section.
